# Lovastatin‐Mediated Changes in Human Tendon Cells

**DOI:** 10.1002/jcp.25010

**Published:** 2015-06-23

**Authors:** Maria Kuzma‐Kuzniarska, Hannah R. Cornell, Michael C. Moneke, Andrew J. Carr, Philippa A. Hulley

**Affiliations:** ^1^ Botnar Research CentreInstitute of Musculoskeletal SciencesNuffield Department of OrthopaedicsRheumatology and Musculoskeletal SciencesUniversity of OxfordOxfordUnited Kingdom

## Abstract

Statins are among the most widely prescribed drugs worldwide. Numerous studies have shown their beneficial effects in prevention of cardiovascular disease through cholesterol‐lowering and anti‐atherosclerotic properties. Although some statin patients may experience muscle‐related symptoms, severe side effects of statin therapy are rare, primarily due to extensive first‐pass metabolism in the liver. Skeletal muscles appear to be the main site of side effects; however, recently some statin‐related adverse effects have been described in tendon. The mechanism behind these side effects remains unknown. This is the first study that explores tendon‐specific effects of statins in human primary tenocytes. The cells were cultured with different concentrations of lovastatin for up to 1 week. No changes in cell viability or morphology were observed in tenocytes incubated with therapeutic doses. Short‐term exposure to lovastatin concentrations outside the therapeutic range had no effect on tenocyte viability; however, cell migration was reduced. Simvastatin and atorvastatin, two other drug family members, also reduced the migratory properties of the cells. Prolonged exposure to high concentrations of lovastatin induced changes in cytoskeleton leading to cell rounding and decreased levels of mRNA for matrix proteins, but increased BMP‐2 expression. Gap junctional communication was impaired but due to cell shape change and separation rather than direct gap junction inhibition. These effects were accompanied by inhibition of prenylation of Rap1a small GTPase. Collectively, we showed that statins in a dose‐dependent manner decrease migration of human tendon cells, alter their expression profile and impair the functional network, but do not inhibit gap junction function. J. Cell. Physiol. 230: 2543–2551, 2015. © 2015 The Authors. *Journal of Cellular Physiology* Published by Wiley Periodicals, Inc.

Statins are a cholesterol‐lowering drug family prescribed to reduce the risk of cardiovascular disease. By inhibiting 3‐hydroxy‐3‐methylglutaryl coenzyme A (HMG‐CoA) reductase, they supress the conversion of HMG‐CoA to mevalonic acid, an important step in cholesterol biosynthesis. Although cholesterol reduction is thought to be the primary mechanism underlying the benefits of statin therapy, statins have also been shown to act through a lipid‐independent mechanism. They prevent the first step of isoprenoid biosynthetic pathway, consequently reducing coenzyme Q and selenoprotein production, and affecting isoprenylation of proteins such as small GTPases. Many positive effects observed in statin patients are at least in part mediated through a lipid‐independent mechanism (Wang et al., 2008; Sadowitz et al., 2010; Zhou and Liao, 2010; Maji et al., 2013).

Although statins are relatively safe they display some adverse effects, which are dose‐dependent and occur across the whole drug class (Hoffman et al., 2012). Statin‐associated complications may include hepatotoxicity and nephrotoxicity; however, muscle‐related side effects are most common. They mainly involve myalgia and myopathy. Severe adverse effects like rhabdomyolysis resulting in skeletal muscle injury are extremely rare (Maji et al., 2013; Ahmad, 2014). The mechanism underlying muscle‐specific side effects has not been fully characterised; however, cholesterol‐independent action of statins is believed to play a role (Maji et al., 2013; Mosshammer et al., 2014). Although skeletal muscles appear to be the most important site of statin‐induced manifestations, lately some potential adverse effects were also reported in tendon (Pullatt et al., 2007; Marie et al., 2008; Beri et al., 2009). Tendinous complications account for about 2% of overall statin‐related adverse effects and include tendinopathy and tendon rupture (Pullatt et al., 2007; Marie et al., 2008; Beri et al., 2009).

Tendons act as force transmitters between muscle and bone. They consist of bundles of collagen with cells (tenocytes) aligned in longitudinal rows separated by collagen fibres. Tenocytes are responsible for the formation and turnover of the extracellular matrix, including the major structural component of tendon, collagen I (Benjamin et al., 2008). They develop organised actin stress fibres aligned along the line of principal strain (Ralphs et al., 2002) as they form a network of longitudinal and lateral cell processes linked by gap junctions (McNeilly et al., 1996). Gap junctions enable intercellular communication between tenocytes and most likely co‐ordinate the activities of individual cells in the tendon. However, their precise role has not been sufficiently studied. Recently, we showed that fluorescence recovery after photobleaching (FRAP) can be used to measure gap junction function in human tendon cells (Kuzma‐Kuzniarska et al., 2014).

The mechanism behind tendon‐specific adverse effects in statin patients is unknown. All proposed mechanisms of tendon injury following statin treatment are hypothetical and based solely on muscle literature (Pullatt et al., 2007; Marie et al., 2008). The only two studies investigating tendon‐related adverse effects of statin therapy were performed in rats and concentrated on the assessment of biochemical and biomechanical changes (de Oliveira et al., 2013, 2015). The aim of this study was therefore to explore tendon‐specific effects of statins in human primary tendon cells.

## Materials and Methods

### Human tenocyte culture

Samples of normal hamstring tendon were obtained from patients, average age 30 (19–45y), undergoing anterior cruciate ligament reconstruction with informed consent and in full compliance with UK HTA and MREC requirements and with the Helsinki Declaration of 1975, as revised in 1983. Tenocytes were derived by standard explant protocol (Torricelli et al., 2006). They were expanded in DMEM/F12 (Lonza, Cheltenham, UK) containing 10% foetal calf serum (FCS) (Biosera, Ringmer, UK) and maintained in DMEM/F12 5% FCS during statin treatment. Tenocytes were treated with 0.2–2 μM lovastatin (Sigma, Poole, UK) dissolved in dimethyl sulfoxide (DMSO) (Sigma) for different times. Furthermore the cells were incubated in the presence of 0.08 and 0.8 μM atorvastatin (Sigma) and 0.048 and 0.48 μM simvastatin (Sigma). The latter was dissolved before treatment in NaOH in ethanol. To reverse lovastatin‐induced changes 100 μM mevalonate (Sigma) was added back. Vehicle controls included DMSO for lovastatin and atorvastatin, NaOH in ethanol for simvastatin and ethanol for mevalonate. Cells were used at passage 4 or below as it has been reported that tenocytes may dedifferentiate at higher passages (Yao et al., 2006).

### Cell viability

The viability of human tenocytes in presence of different statin concentrations was assessed using LIVE/DEAD® kit (Life Technologies, Paisley, UK). To distinguish between viable and dead cells tenocytes were co‐stained with 4 µM calcein acetoxymethylester (AM) solution and 4 µM ethidium homodimer‐1 (EthD‐1) for 15 min at room temperature in serum free medium. In live cells nonfluorescent calcein AM undergoes conversion by intracellular esterases to fluorescent calcein, at the same time EthD‐1 enters only cells with damaged membranes and stains the dead cell nuclei. Images were acquired using Nikon TE300 microscope (Nikon Instruments, Tokyo, Japan) with Retiga CCD camera (QImaging, British Columbia, Canada). To quantify the viability of cells we used alamarBlue® assay (Life Technologies). Tenocytes were seed in 96‐well plates and cultured in presence of different concentration of lovastatin for 1, 3 and 7 days. Subsequently the cells were incubated with alamarBlue for 3 h at 37°C and fluorescence intensity was measured using FLUOstar Optima (BMG LABTECH, Ortenberg, Germany). The amount of fluorescence corresponds to metabolic activity and is proportional to the number of viable cells.

### Migration assay

Cells were grown to confluence in 24‐well plates and pre‐treated with different concentrations of statins for 2 days. Subsequently a 10–200 μl pipette tip was used to scratch a wound through the centre of the well. For each condition at total of 6 bright field images from 2 wells for each donor was analysed. Images were acquired immediately at 0 h and at 16 h after the initial scratch was performed using Nikon TE300 microscope. The drug was present in the medium throughout the assay. To calculate the changes in width of the wound ImageJ software was used.

### Fluorescence recovery after photobleaching (FRAP)

The cells were grown to confluency, loaded for 15 min with 4 µM calcein AM at room temperature in serum‐free medium, subsequently washed several times with pre‐warmed medium. Zeiss LSM 710 scanhead (Zeiss GmbH, Jena, Germany) coupled to an inverted Zeiss Axio Observer.Z1 microscope (Zeiss GmbH) was used for photobleaching and fluorescence imaging. FRAP was performed as described previously (Kuzma‐Kuzniarska et al., 2014). In brief, a region of interest (ROI) was manually drawn around whole fluorescent cells selected for bleaching and subsequently bleached at 100% laser power. Next a time‐lapse series was taken to record calcein recovery every 5 sec up to 4 min. To further monitor the changes in recovery, a reference unbleached cell and background, were analysed simultaneously. At least 10 cells were photobleached and imaged for each condition. Mobile fraction percentage was used to quantify the differences. The only source of fluorescent calcein in this experiment is the neighbouring cells as the dye is not present in the medium during acquisition. Cells that either do not have gap junctions or their gap junctions have been blocked by inhibitors are unable to recover after photobleaching (Kuzma‐Kuzniarska et al., 2014).

### F‐actin staining

Cells were grown on coverslips, fixed with 10% formalin for 30 min and subsequently incubated with 0.1% Triton X‐100 in PBS for 10 min at room temperature. F‐actin staining was performed using rhodamine‐phalloidin solution (Life Technologies) according to manufacturer's instruction. Images were acquired using Nikon TE300 microscope.

### QPCR

Following standard mRNA extraction (Liang et al., 2008), QPCR was performed using a Rotorgene 3000 and Quantitect Sybr Green with Quantitect primer assays: TBP (QT00000721), BMP‐2 (QT00012544), COL1A1 (QT00037793), COL3A1 (QT00058233). Reagents were from Qiagen (Hilden, Germany). Melt curve analysis confirmed a single product from each reaction. Absence of genomic DNA was confirmed using “no RT“ controls. Samples (run in duplicate) were analysed by comparative quantification (Pfaffl, 2001) using Rotorgene software version 1.7. TATA box binding protein (TBP) was compared with GAPDH and selected for normalization of gene expression based on greater insensitivity to lovastatin.

### Western blotting

Western blotting was performed as described previously (Liang et al., 2008). Antibodies: Unprenylated‐Rap1a (sc‐1482, Santa Cruz Biotechnology, Inc, Santa Cruz, CA), GAPDH (G9545, Sigma), anti‐goat‐HRP (Dako, Glostrup, Denmark) and anti‐rabbit‐HRP (Abcam, Cambridge, UK). Signal was captured using the Chemic Doc‐It Imaging System (UVP, Upland, CA) and VisionWorks LS software (VisionWorks LS v 6.7.4, UVP).

### Statistical analysis

All experiments have been carried out on cells from at least 3 different tissue donors. Statistics were performed using GraphPad Prism version 5.0b (Mac OS X) (GraphPad Software, San Diego, CA). A *P*‐value lower than 0.05 was considered statistically significant.

## Results

### Effect of different concentrations of lovastatin on human tenocyte viability

**Figure 1:**
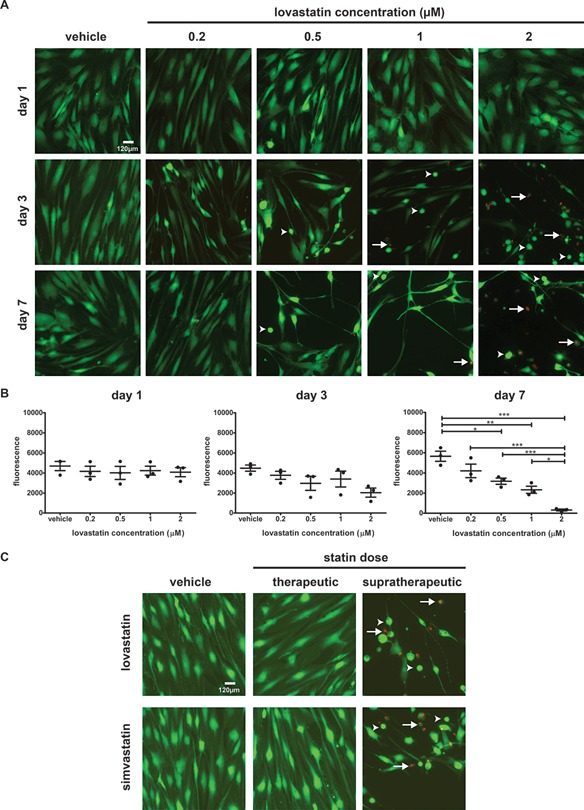
Effect of different concentrations of statins on tenocyte viability. Human tendon cells were cultured in presence of different concentrations of statins for up to 7 days. (A) Representative images of live (green) and dead (red, arrows) stained cultures treated with different concentrations of lovastatin for 1, 3 and 7 days. Note the cell rounding (arrowheads). (B) To quantify the viability of lovastatin‐treated cells alamarBlue was used. The results show mean fluorescence intensity ± SE from 3 donors. One‐way ANOVA with Tukey‘s test was performed. *P < 0.05, **P < 0.01, ***P < 0.001. (C) Representative images of live and dead stained human tenocytes cultured in presence of 0.2 µM (therapeutic) and 2 µM (supratherapeutic) lovastatin and 0.048 µM (therapeutic) and 0.48 µM (supratherapeutic) simvastatin for 7 days. Note the presence of numerous rounded but live stained cells (arrowheads).

In order to investigate the effects of lovastatin on the viability of human tenocytes, the cells were incubated with different concentrations of the drug and subsequently co‐stained with calcein (live) and EthD‐1 (dead). No dead cells were detected in any of the conditions tested following 1 day of lovastatin treatment (Fig. 1A). Furthermore no changes in viability were observed in cells treated up to 1 week with 0.2 μM lovastatin, a dose within the therapeutic range of statins (Cho et al., 2011) (Fig. 1A). Starting from day 3 the drug at higher concentrations caused some cells to round up in accordance with well‐described effects of lovastatin on cell morphology and cytoskeleton (Fenton et al., 1992). The round cells were viable as they were strongly positive for calcein live stain and were not stained with EthD‐1 (Fig. 1A, arrowheads). At the same time some dead cells were detected in the cultures treated with higher concentration of lovastatin for 3 days or longer (Fig. 1A, arrows). To quantify the effects, tenocytes were exposed to increasing concentrations of lovastatin and analysed using alamarBlue. The fluorescence intensity of alamarBlue is directly proportional to the metabolic activity of the cells and can be used as viability indicator. As shown in the Figure 1B no changes in fluorescence were observed after a short‐term exposure to lovastatin at any concentration tested. Similarly at day 3 no significant reduction in viability was noted but a non‐statistical decrease in fluorescence was observed at higher doses. At 1 week the viability of tenocytes was compromised in all conditions in a dose‐dependent manner reaching statistical significance at 0.5–2 μM concentration (Fig. 1B). It is important to note that lovastatin inhibits cell proliferation (Fenton et al., 1992) therefore the observed decrease in fluorescence does not reflect only the cytotoxic but also the cytostatic effect of the drug on tendon cells. Finally, we compared the viability of tendon cells in presence of lovastatin and simvastatin, a derivative of lovastatin linked to higher risk of adverse effects (Hoffman et al., 2012). The therapeutic and supratherapeutic dose for both statins was selected based on the literature (Novakova et al., 2009; Cho et al., 2011). As demonstrated in the Figure 1C simvastatin, similarly to lovastatin, caused rounding up of cells and reduction in viability at supratherapeutic dose only.

### Lovastatin dose‐dependently decreases migration of human tenocytes

**Figure 2:**
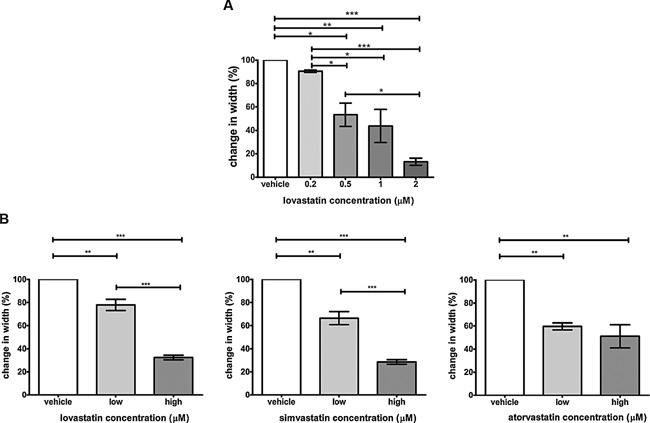
Statins decrease migration of human tenocytes in dose‐dependent manner. Tenocytes pre‐treated with different concentrations of statins for 2 day were subjected to a scratch assay. The migration was evaluated after 16h. The drug was present in the medium during this time. (A) The graph shows the change in average width of the scratch between conditions normalised to the vehicle control. The results show mean ±SE from 3 donors. One‐way ANOVA with Tukey‘s test was performed. *P < 0.05, **P < 0.01, ***P < 0.001. (B) Comparison of migratory properties of human tenocytes treated with therapeutic and supratherapeutic doses of lovastatin, simvastatin and atorvastatin using a scratch assay. Following concentrations were used: 0.2 µM (low) and 2 µM (high) lovastatin, 0.048 µM (low) and 0.48 µM (high) simvastatin, 0.08 µM (low) and 0.8 µM (high) atorvastatin. Results were analyzed using two‐tailed, unpaired Student's *t*‐test and are shown as a mean ± SE of 3 donors. Statistical differences are indicated as **P < 0.01, ***P < 0.001.

Confluent cultures of human tenocytes were pre‐treated with different concentrations of lovastatin for 2 days before they were subjected to a scratch assay. As shown in the Figure 2A lovastatin decreased migration of tenocytes in a dose‐dependent manner. While lovastatin had no significant effect on tenocyte viability at day 3 (Fig. 1), migratory response of the cells was affected. The impairment in migration was observed in all conditions tested and reached statistical significance at 0.5–2 μM concentration. Ultimately, we investigated the migratory capacity of tenocytes in presence of three different members of statin family, namely lovastatin, simvastatin and atorvastatin. Statin concentrations were selected based on the literature (Novakova et al., 2009; Cho et al., 2011). All statins tested significantly reduced migration of tenocytes at the therapeutic (low) and supratherapeutic (high) dose, confirming the negative effect of statins on migration.

### Lovastatin regulates gene expression in human tenocytes

**Figure 3:**
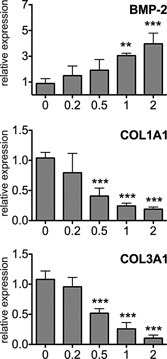
Effect of different concentrations of lovastatin on tenocyte expression profile. Tenocytes were cultured with vehicle control, 0.2, 0.5, 1 or 2 μM lovastatin for 1 week. *BMP‐2*, *COL1A1* and *COL3A1* mRNA levels were assessed by QPCR and normalised to *TBP*. One‐way ANOVA with Dunnett post‐hoc was performed. The results are shown as mean ± SD of 4 donors. **P < 0.01, ***P < 0.001.

Previously a chronic treatment with simvastatin and atorvastatin has been shown to reduce collagen I expression in rat Achilles tendons (de Oliveira et al., 2013). To evaluate the effect of statins on gene expression in human tenocytes, the cells were treated with 0.2–2 μM lovastatin for 1 week. Accordingly lovastatin dose‐dependently decreased collagen I (*COL1A1*) and collagen III (*COL3A1*) mRNA levels showing a significant down‐regulation at 0.5–2 μM concentration. Interestingly, at the same time mRNA levels of bone morphogenetic protein‐2 (*BMP‐2*), an osteogenic growth factor, were dose‐dependently increased in tenocytes treated with lovastatin (Fig. 3) as previously reported for osteoblasts (Mundy et al., 1999).

### Lovastatin affects gap junction‐mediated intercellular communication in human tenocytes

**Figure 4:**
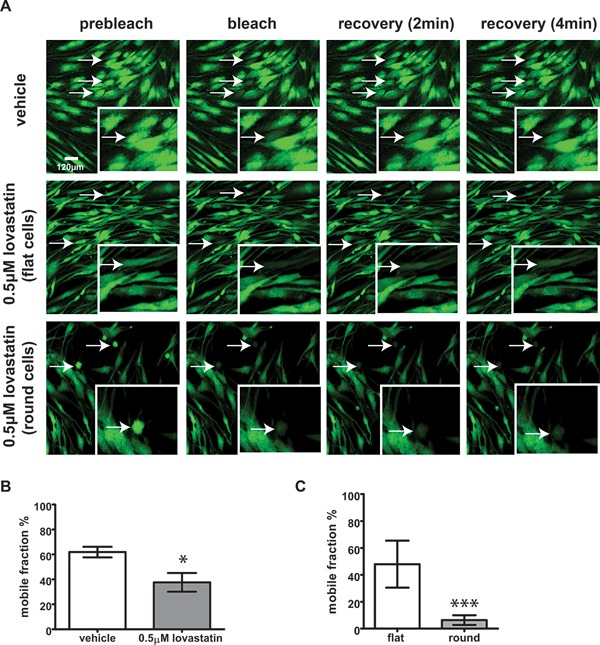
Lovastatin‐induced rounding of cells leads to a decrease in gap junctional communication in human tenocytes. Cells were treated with 0.5 μM lovastatin for 1 week, labelled with calcein and subjected to FRAP analysis. (A) An example of a time‐lapse series recorded for FRAP. Arrows point towards bleached cells. (B) The mobile fraction percentage in untreated and lovastatin‐treated tendon cells. Note two populations of calcein‐labelled cells in lovastatin‐treated cultures: flat and round. Results were analyzed using two‐tailed, unpaired Student's t‐test and are shown as a mean ± SE of 3 donors. Statistical differences are indicated as *P < 0.05. (C) A comparison between gap junctional communication in flat and round cells in a culture from the same patient. Results shown are mean ± SD.

In order to assess intercellular communication in tenocytes treated with statins, cells were incubated for 1 week in presence of 0.5 μM lovastatin, loaded with calcein and subjected to photobleaching, according to our FRAP protocol for measuring gap junction function in human tenocytes (Kuzma‐Kuzniarska et al., 2014). A time‐lapse series was then taken to record fluorescence recovery. As shown in the Figure 4A, cells incubated with the vehicle rapidly re‐acquired the fluorescent dye from adjacent cells, while cells treated with lovastatin demonstrated a decreased recovery suggesting impairment in gap junction‐mediated communication. Accordingly mobile fraction percentage reached about 60% in control cells and 37% in lovastatin‐treated cultures (*P* < 0.05) (Fig. 4B). Since two populations of tenocytes can be distinguished in lovastatin‐treated cultures, cells that retain their fibroblastic morphology (flat) and cells that undergo rounding up (round), we decided to analyse these two populations separately. As demonstrated in the Fig. 4C the intercellular communication was dramatically reduced in round cells (6%) when compared to flat cells (47%).

### Lovastatin‐induced changes in tenocytes are accompanied by changes in F‐actin

**Figure 5:**
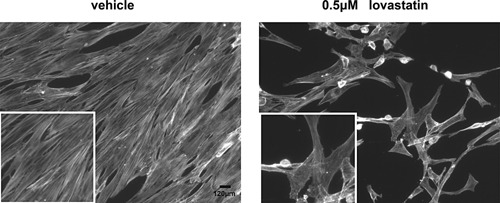
Lovastatin‐induced changes in morphology are accompanied by changes in F‐actin. The cells were grown in presence of 0.5 μM lovastatin for 1 week. Representative images of F‐actin stained tenocyte cultures are shown.

Human tenocytes demonstrate highly organized actin stress fibres that extend the entire length of the cell (Ralphs et al., 2002). Lovastatin disrupts actin cytoskeleton causing cell rounding (Fenton et al., 1992). Consequently we performed phalloidin staining to confirm changes in cytoskeleton in tendon cells treated with lovastatin. As shown in the Figure 5 treatment with 0.5 μM lovastatin resulted in disruption of actin organization. Adherent cells that retained a fibroblastic morphology contained less stress fibres and the remaining fibres were mainly located in the periphery of the cell. No organised actin fibres were observed in round cells.

### Lovastatin‐mediated changes correlate with alterations in prenylation status of Rap1a

**Figure 6:**
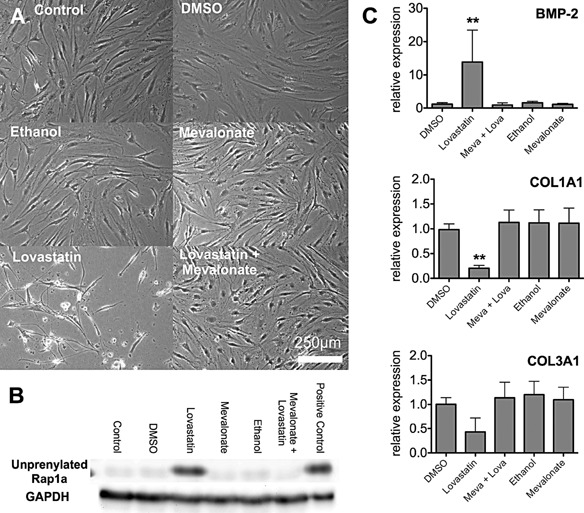
Mevalonate prevents lovastatin‐induced changes in cell morphology, prenylation status and gene expression. Tenocytes were cultured with medium alone, vehicle control (DMSO or ethanol), 100μM mevalonate, 0.5 μM lovastatin or lovastatin and mevalonate (Meva + Lova) for 1 week. (A) Bright field images demonstrating cell morphology are presented. (B) Unprenylated Rap1a was detected in lovastatin cultures using Western blotting. (C) *BMP‐2*, *COL1A1* and *COL3A1* mRNA levels were assessed by QPCR and normalised to *TBP*. One‐way ANOVA with Tukey post‐hoc was performed. The results are shown as mean ± SD of 4 donors. **P < 0.01.

Statin‐mediated changes are believed to result from inhibition of HMG‐CoA reductase, with consequent reduction in prenylation of small GTPases. To confirm that observed changes in tendon cells were due to inhibition of HMG‐CoA reductase by lovastatin, add‐back experiments were performed using an excess of the downstream metabolite mevalonate. As shown in Figure 6A lovastatin treatment caused cell rounding. Mevalonate prevented this, maintaining the fibroblastic morphology of the tenocytes. Western blotting for the unprenylated form of the small GTPase Rap1a confirmed that lovastatin at 0.5 μM concentration inhibited protein prenylation (Fig. 6B). Finally, mevalonate restored protein prenylation (Fig. 6B) and prevented the lovastatin‐induced decrease in *COL1A1* (*P* < 0.01) and *COL3A1* mRNA and increase in *BMP‐2* (*P* < 0.01) (Fig. 6C).

## Discussion

Several studies have reported statin‐associated manifestations in tendons (Pullatt et al., 2007; Marie et al., 2008; Beri et al., 2009). Although tendon‐specific side effects are still considered to be relatively rare, the number of patients experiencing tendon‐related manifestations has been constantly increasing in parallel with the rise in the statin prescriptions (Marie et al., 2008). Proposed mechanisms of statin‐induced tendon injury are based on muscle literature and include destabilisation of cell membrane through reduction in cholesterol content, tenocyte apoptosis leading to tendon damage as well as impairment of tendon remodelling due to changes in MMP expression and activity (Pullatt et al., 2007; Marie et al., 2008). So far only one study has investigated the mechanism underlying tendon‐specific adverse effects. Accordingly de Oliveira et al. showed that chronic treatment with statins induce changes in collagen I expression and MMPs activity in rat tendons (de Oliveira et al., 2013). The exact mechanism behind the tendon‐specific adverse effects still remains unknown.

In this study for the first time we have addressed the effects of statins on human tendon cells. Accordingly we showed that lovastatin alters cell shape, density, migration and expression profile of human tenocytes but does not directly stop intercellular communication. We demonstrated that even a short‐term exposure to statins can decrease migratory properties of tenocytes. Tenocyte motility is essential for tendon healing as the cells have to migrate to the site of injury to produce extracellular matrix and subsequently remodel the tissue (Sharma and Maffulli, 2006). An inhibitory effect of statins on migration has been described previously in smooth muscle cells (Porter et al., 2002; Shen et al., 2010), rheumatoid fibroblast‐like synoviocytes (Xiao et al., 2013) and cardiac fibroblasts (Copaja et al., 2012).

It has been suggested that statin‐induced inhibition of migration might be mediated by gap junctions. Rat aortic smooth muscle cells (RASMCs) incubated with increasing concentrations of lovastatin showed dose‐dependent inhibition in gap junction‐mediated intercellular communication (Shen et al., 2010). In our study we demonstrated that statin treatment impairs intercellular communication also in human tenocytes. This finding is in agreement with previous studies showing reduction in gap junction proteins Cx43 and Cx40 expression following administration of statins (Kwak et al., 2003; Wang et al., 2005). However gap junction‐mediated intercellular communication in tendon cells also strongly depends on number of cell‐to‐cell contacts (Kuzma‐Kuzniarska et al., 2014). We showed that reduction in communication is at least in part caused by reduction in cell‐to‐cell contact due to reduced cell density and spreading or increased rounding induced by lovastatin. Ultimately, it is important to note that in our study as well as in the study on RASMCs only concentration higher than the therapeutic dose of the drug lead to significant decrease in gap junctional communication in the cells.

It is well accepted that inhibition of HMG‐CoA reductase by lovastatin results in suppression of protein prenylation, changes in microfilaments followed by rounding of cells as well as inhibition in proliferation though cell arrest in G1 and at high doses cytotoxic effect. In addition mevalonate, a downstream metabolite in the cholesterol biosynthesis pathway that statins inhibit, is able to reverse statin‐induced changes (Jakobisiak et al., 1991; Fenton et al., 1992). In our study we confirmed that lovastatin at higher concentration causes rounding of tenocytes, which is accompanied by the disruption of cytoskeleton. Furthermore, we showed that lovastatin inhibits prenylation, using small GTPase Rap1a as an example. Although many small GTPases will be affected and could be of interest, Rap1 is a Ras‐like small GTPase that plays a crucial role in cell‐cell junction formation (Kooistra et al., 2007). Rap1 is also essential for cell motility thus silencing of Rap1a and/or Rap1b results in significant reduction of migration in endothelial cells (Carmona et al., 2009). This is in agreement with our data showing reduction in migration of tenocytes in presence of lovastatin. Finally, we demonstrated that human tenocytes treated with lovastatin in presence of mevalonate were able to regain fibroblastic morphology and restore protein prenylation of Rap1a.

The extracellular matrix of the tensional region of tendons is composed mostly of collagen I with small quantities of other collagens including collagen III (Riley, 2004). Previously statin treatment was demonstrated to decrease collagen I and III expression in dermal (Louneva et al., 2006) and kidney fibroblasts (Ikeuchi et al., 2004). Also the latest study by de Oliveira et al. showed that a low dose of atorvastatin can reduce collagen I expression in tendons of rats subjected to chronic statin treatment (de Oliveira et al., 2013). Here, we reported that statin treatment decreases both *COL1A1* and *COL3A1* mRNA in human tendon cells. GGTI‐298, a specific inhibitor of geranylgeranyl transferase I (GGTase I) that prenylates the Rho and Rac small GTPases, was demonstrated to decrease collagen expression in dermal fibroblasts (Rosenbloom et al., 2000), whereas RhoQ63L, a constitutively active RhoA mutant, was shown to increase the expression (Novakofski et al., 2009). These data strongly implicate the small GTPases, particularly RhoA, in the control of tenocytic collagen matrix production. Finally, we showed that treatment with lovastatin increases *BMP‐2* expression in human tenocytes. Within the musculoskeletal field statins were originally reported as osteogenic agents that induced expression of BMP‐2 in osteoblasts (Mundy et al., 1999). Whether the increase in *BMP‐2* may be indicative of a phenotypic switch in statin‐treated tendon cells remains however to be investigated.

In this study we examined the effects of statins on migration, gap junction function, actin cytoskeleton and expression profile of human tendon cells. Further we showed that statins can be used as biochemical tools for exploring prenylation‐sensitive pathways in tenocytes in vitro. Collectively, these findings deliver novel insight into tenocyte biology and identify pathways that may contribute to tendon‐specific adverse effects in statin therapy. Further studies are required to investigate the effects of chronic exposure to statins in human tenocytes.
